# Sirtuin 1 Inhibiting Thiocyanates (S1th)—A New Class of Isotype Selective Inhibitors of NAD^+^ Dependent Lysine Deacetylases

**DOI:** 10.3389/fonc.2020.00657

**Published:** 2020-04-30

**Authors:** Nathalie Wössner, Zayan Alhalabi, Jessica González, Sören Swyter, Jin Gan, Karin Schmidtkunz, Lin Zhang, Alejandro Vaquero, Huib Ovaa, Oliver Einsle, Wolfgang Sippl, Manfred Jung

**Affiliations:** ^1^Department of Pharmaceutical and Medicinal Chemistry, Institute of Pharmaceutical Sciences, University of Freiburg, Freiburg im Breisgau, Germany; ^2^Department of Medicinal Chemistry, Institute of Pharmacy, University of Halle-Wittenberg, Halle, Germany; ^3^Chromatin Biology Laboratory, Josep Carreras Leukaemia Research Institute (IJC), Badalona, Barcelona, Spain; ^4^Department of Cell and Chemical Biology, Oncode Institute, Leiden University Medical Center, Leiden, Netherlands; ^5^Department of Protein Crystallography, Institute of Biochemistry, University of Freiburg, Freiburg im Breisgau, Germany

**Keywords:** sirtuins, lysine deacetylase, thiocyanate, DNA damage, histone, H2AX, p53

## Abstract

Sirtuin 1 (Sirt1) is a NAD^+^ dependent lysine deacetylase associated with the pathogenesis of various diseases including cancer. In many cancer types Sirt1 expression is increased and higher levels have been associated with metastasis and poor prognosis. However, it was also shown, that Sirt1 can have tumor suppressing properties and in some instances even a dual role for the same cancer type has been reported. Increased Sirt1 activity has been linked to extension of the life span of cells, respectively, organisms by promoting DNA repair processes and downregulation of tumor suppressor proteins. This may have the downside of enhancing tumor growth and metastasis. In mice embryonic fibroblasts depletion of Sirt1 was shown to decrease levels of the DNA damage sensor histone H2AX. Impairment of DNA repair mechanisms by Sirt1 can promote tumorigenesis but also lower chemoresistance toward DNA targeting therapies. Despite many biological studies, there is currently just one small molecule Sirt1 inhibitor in clinical trials. Selisistat (EX-527) reached phase III clinical trials for treatment of Huntington's Disease. New small molecule Sirt1 modulators are crucial for further investigation of the contradicting roles of Sirt1 in cancer. We tested a small library of commercially available compounds that were proposed by virtual screening and docking studies against Sirt1, 2 and 3. A thienopyrimidone featuring a phenyl thiocyanate moiety was found to selectively inhibit Sirt1 with an IC_50_ of 13 μM. Structural analogs lacking the thiocyanate function did not show inhibition of Sirt1 revealing this group as key for the selectivity and affinity toward Sirt1. Further analogs with higher solubility were identified through iterative docking studies and *in vitro* testing. The most active compounds (down to 5 μM IC_50_) were further studied in cells. The ratio of phosphorylated γH2AX to unmodified H2AX is lower when Sirt1 is depleted or inhibited. Our new Sirtuin 1 inhibiting thiocyanates (S1th) lead to similarly lowered γH2AX/H2AX ratios in mouse embryonic fibroblasts as Sirt1 knockout and treatment with the reference inhibitor EX-527. In addition to that we were able to show antiproliferative activity, inhibition of migration and colony forming as well as hyperacetylation of Sirt1 targets p53 and H3 by the S1th in cervical cancer cells (HeLa). These results reveal thiocyanates as a promising new class of selective Sirt1 inhibitors.

## Introduction

Sirtuins are deacylases able to cleave off acetyl and also longer chain acyl groups from the ε-amino residue of lysines in histones and non-histone proteins in a NAD^+^-dependent manner. For their activity on histones they have been designated to form class III of the histone deacetylases (HDACs). Generally, HDACs are known to deacetylate resp. deacylate various protein substrates and for sirtuins many findings actually focus on non-histone substrates (1). The human genome contains seven sirtuins isotypes (Sirt1-7) that differ in substrate spectrum and localization (2). With regard to drug discovery the majority of efforts has focused on Sirt2 where many small molecule inhibitors are now present. Other isotypes have been addressed as well but only for Sirt1 a drug is currently in clinical trials (see below) (3, 4). Due to conflicting evidence of roles of sirtuin isotypes in different diseases both activators and inhibitors have been investigated (5–7).

Sirt2 has been discussed as an anticancer target but also tumor suppressive activities of Sirt2 have been mentioned (8–15). These effects might be tissue or organ specific which complicates drug development and no Sirt2 inhibitor has reached clinical trials yet. Sirt6 has been identified as a tumor suppressor and hence Sirt6 activators are the focus of drug discovery efforts regarding this phenotype (16, 17). But also tumor promoting effects of Sirt6 have been described (18). In addition, Sirt6 inhibition might be a way to increase the efficacy of cytostatic drugs with DNA-damaging mode of action (19).

**Figure 1:**
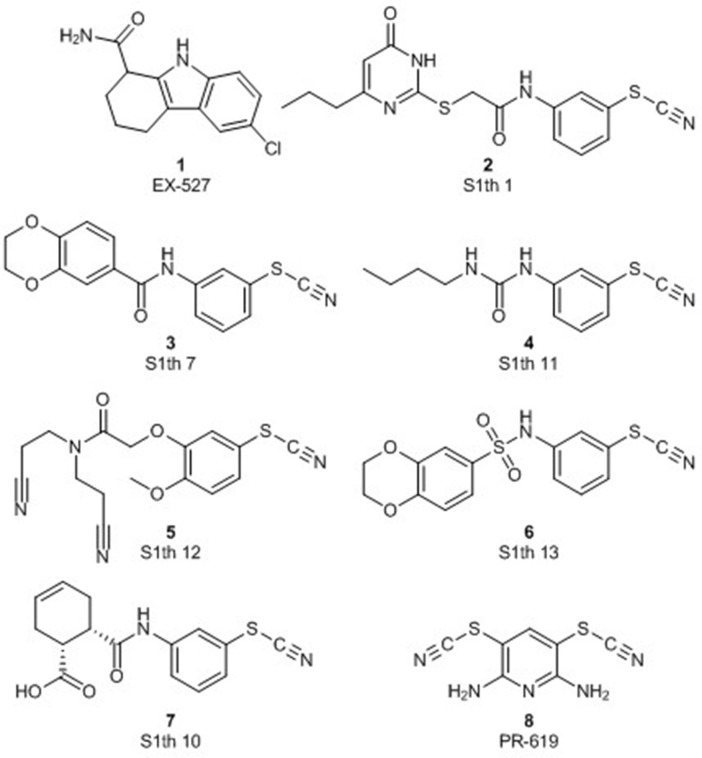
Structures of **S1th 1**, **7**, **S1th 10–13**, the pan-DUB inhibitor **PR-619** and the reference Sirt1 inhibitor **EX-527**.

Regarding Sirt1, it has been studied strongly in the context of neurodegenerative diseases and Selisistat [EX-527 (**1**), Figure 1] is a potent and selective Sirt1 inhibitor that was undergoing clinical testing in Huntington's disease (20). A strong focus attention was dedicated to potentially lifespan extending Sirt1 activators. The initial studies mostly dealt with resveratrol, a natural product with pleiotropic activities which makes mechanistic studies difficult. Later, drug-like sirtuin activating compounds (STACs) were identified and went into clinical trials. While the discussion on the relevance and robustness of activation of deacetylation by Sirt1 has been very controversial, some effects of resveratrol have indeed been linked to Sirt1 in animal models and depending on the deacetylation substrate, activation has been proven in reliable biochemical assays (21). With regard to cancer, Sirt1 was postulated as an anticancer target, e.g., promoting epithelial-to-mesenchymal transition (EMT) in many cancer types (22) but it was also reported to suppress EMT in types like ovarian cancer. Yang et al. postulated that the conflicting behavior of Sirt1 in cancer cells may depend on its subcellular localization (23). In some cancer types both tumor promoting and suppressing actions have been described, for example in prostate cancer. In Sirt1 knockout mice increased cell proliferation of prostatic intraepithelial neoplasia was observed, implicating Sirt1 as a tumor suppressor (24). However, via global transcriptome analysis increased levels of Sirt1 were identified as a key biomarker for prostate cancer suggesting a tumor promoting influence of Sirt1 (25). Several important tumor suppressors like p53, FOXO3a, or E2F1 that induce apoptosis in malignant cells (e.g., in breast cancer) are deacetylated by Sirt1, and thereby inactivated, promoting cell survival (26–29). In breast cancer tissue elevated Sirt1 expression correlates with tumor size, high histological grades and lymph node metastasis (30). Nevertheless, Sirt1 can still act as a tumor suppressor in breast cancer cells as well. It is crucial for DNA damage response, regulates several DNA repair enzymes like Ku70 and thereby enables stable, efficient DNA repair (31, 32). Some known oncogenes like NF-κB are directly deacetylated by Sirt1 promoting downregulation of the NF-κB-dependent cell survival pathway (33).

In some cancer types (e.g., glioma, bladder, or ovarian cancer) lower expression levels of Sirt1 have been detected, although in most cancer types an increased expression was observed (34). A meta-analysis showed that Sirt1 overexpression significantly correlates with poor prognosis in solid tumors (35). Anticancer effects of sirtuin inhibitors have been described on a cellular level. In cervical cancer cells EX-527 induced cell death while inhibition of the isotype Sirt2 led to cell cycle arrest. In the breast carcinoma cell line MCF-7 though, the opposite effect was observed, Sirt1 inhibition by EX-527 led to cell cycle arrest while treatment with Sirtinol or Salermide (Sirt1/2 inhibitors with a stronger effect on Sirt2) resulted in cell death (36, 37). In melanoma, chronic lymphocytic leukemia as well as hepatocellular carcinoma cell lines both Sirt1 inhibitors (EX-527) and Sirt2 inhibitors impaired cell growth and viability (38–40).

As already mentioned, Sirt1 plays a pivotal role in DNA damage response (DDR). For example, phosphorylation of the DNA damage sensor H2AX which gets phosphorylated to γH2AX upon double strand breaks (DSBs) in healthy cells is significantly downregulated when Sirt1 is inhibited or depleted (41, 42). DDR can be regulated through Sirt1 either by direct histone deacetylation which changes chromatin compaction or by activation and inactivation of non-histone proteins that are involved in the major DNA repair mechanisms: homologous recombination (HR), non-homologs end joining (NHEJ), nucleotide excision repair (NER), mismatch repair (MMR), and base excision repair (BER) (43–47). Due to Sirt1s implications in DNA damage sensing and recruitment of repair proteins it can alter resistance toward some cancer therapies targeting DNA stability. Application of EX-527 reduced chemoresistance in endometrial carcinoma cells (48).

EX-527 is widely used for studies on the effects of Sirt1 inhibition in cells and living organisms, partially due to a lack of other selective Sirt1 inhibitors. The only two compounds that show similar potency toward Sirt1 are its analog CHIC-35 and the Suramin analog NF675 (49, 50). However, both of them show slightly less selectivity over Sirt2 (51) and Suramins are usually poorly cell permeable. Other than that, only few specific Sirt1 inhibitors have been identified. Several examples with low micro molar affinity exist, like certain Splitomicin derivatives or the so-called spiro series (52, 53). To get more insights into the role of Sirt1 in different cancer types and to better examine its therapeutic potential in cancer further Sirt1 inhibitors are needed. Here we present a new class of sirtuin 1 inhibitors based on a specific interaction with a thiocyanate moiety that lead to altered γH2AX/H2AX ratios in mouse embryonic fibroblast cells.

## Materials and Methods

All test compounds are commercially available and were purchased from Princeton BioMolecular Research, Sigma Aldrich, Enamine, or Chembridge and used as received.

### Enzyme Expression and Purification

Recombinant human Sirt5_37−310_ was purchased readily in Tris Buffer [25 mM Tris, 100 mM NaCl, 5 mM DTT, 10% glycerol (v/v)] from Enzo Life Sciences (NY, USA). Human Sirt3_118−395_ was expressed and purified as described previously (54). For expression of human Sirt1_134−747_ and Sirt2_56−356_ chemically competent cells of *E. coli* BL21 Star (DE3) were transformed with the expression vectors pET30S-hSirt1_134−747_ or pET30S-hSirt2_56−356_. Bacteria were grown at 37°C in 2×YT medium supplemented with 50 μg·mL^−1^ of kanamycin to an OD_600_ of 0.6. Then isopropyl-β-D-1-thiogalactopyranoside (IPTG) was added to a final concentration of 1 mM to induce gene expression. After further cultivation at 20°C for 12 h, the cells were harvested by centrifugation for 15 min at 5,000 g. The cells were resuspended in lysis buffer [100 mM Tris/HCl buffer at pH 8.0, 150 mM NaCl and 10% (v/v) glycerol] and disrupted by ultrasonication (Branson Digital Sonifier 250) at 70% amplitude for 10 min (3 s working, 10 s pause). The crude extract was cleared by centrifugation at 100,000 g for 1 h, and the supernatant was loaded onto a Strep-Tactin Superflow cartridge (5 ml bed volume, IBA Lifescience, Germany). Target proteins were eluted with lysis buffer containing 5 mM D-Desthiobiotin (IBA Lifescience, Germany) and further separated by size-exclusion chromatography (Superdex S200 26/60, GE Healthcare, IL, USA) equilibrated in Tris/HCl buffer (20 mM, 150 mM NaCl, pH 8.0). Pure protein was concentrated by ultrafiltration, flash-frozen in liquid nitrogen and stored at −80°C until further use. Identity as well as purity were verified by SDS-PAGE (55) and protein concentration was determined by the bicinchoninic acid (BCA) method, using bovine serum albumin (BSA) as a standard (56). Deacetylase activity was confirmed to be NAD^+^-dependent and could be inhibited with the physiological sirtuin inhibitor nicotinamide.

### *In vitro* Characterization

#### Homogeneous ZMAL-Based Fluorescence Assay for Class I Sirtuins

All compounds were tested in the trypsin-coupled high-throughput ZMAL-assay in black 96-well plates (OptiPlateTM−96F, black, 96 well, Pinch bar design, PerkinElmer, USA), using ZMAL (Z-Lys(acetyl)-AMC) as a substrate (57). Sirt1_134−747_, Sirt2_56−356_, and Sirt3_118−395_ were mixed with 5 μL substrate (10.5 μM final assay concentration, diluted from a 12.6 mM stock in DMSO) and 3 μL Inhibitor in DMSO at various concentrations or DMSO as a control [final DMSO concentration 5% (v/v)]. The mixture was supplemented with assay buffer (50 mM Tris/HCl, 137 mM NaCl, 2.7 mM KCl, 1 mM MgCL_2_, pH 8.0) to a total volume of 55 μL. Enzyme concentration was adjusted to get a final conversion of 20–30%. Addition of 5 μL NAD^+^ (6 mM in assay buffer, final assay concentration of 500 μM) initiated the catalytic reaction and the plates were incubated at 37°C for 4 h with agitation at 140 rpm. The catalytic reaction was stopped by addition of 60 μL stop solution [50 mM Tris, 100 mM NaCl, 6.7% (V/V) DMSO, 5.5 U/μL trypsin, 8 μM nicotinamide, pH 8.0]. The plate was again incubated at 37°C and 140 rpm for 20 min to release free AMC from the deacetylated substrate by trypsin digestion. Afterwards, fluorescence intensity was measured in a microplate reader (λ_Ex_ = 390 nm, λ_Em_ = 460 nm, BMG POLARstar Optima, BMG Labtech, Germany). An enzyme-free blank control and a 100% conversion control containing AMC instead of ZMAL were measured in addition. Inhibition was calculated in % in relation to a DMSO control after blank signal subtraction and IC_50_ values were determined using a non-linear regression to fit the dose-response curve with OriginPro 9G (OriginLab, USA). Pre-tests as well as IC_50_ determination was carried out at least twice in duplicates.

#### Homogeneous ZKsA-Based Fluorescence Assay for Sirt5

Inhibition of Sirt5 was measured using a general procedure described before with small modifications (58). Sirt5 was purchased from Enzo Life Science (NY, USA) and used as received. Z-Lys(succ)-aminomethyl coumarin (ZKsA) was used as a substrate for Sirt5 mediated desuccinylation. In black 384-well non-binding plates (Greiner Bio-One, Monroe, NC) Sirt5 was mixed with 2 μL of ZKsA (1 mM stock solution in assay buffer [(50 mM Tris·HCl, 137 mM NaCl, 2.7 mM KCl, 1 mM MgCl2, pH 8.0, and 0.1% PEG8000), 100 μM final assay concentration], 1 μL of Inhibitor dissolved in DMSO or DMSO as a control [final DMSO concentration 5% (v/v)], 2 μL NAD^+^ (5 mM stock solution in assay buffer, final assay concentration 500 μM) and filled up to a total volume of 20 μL with assay buffer. The mixture was incubated for 1 h at 37°C and 140 rpm before 4 μL of trypsin solution (50 mM Tris, 100 mM NaCl, pH 8.0, 6 mg/mL trypsin buffer, 1 mg/mL final assay concentration) was added to stop the enzymatic reaction. After 2 min of incubation at 37°C and 140 rpm fluorescence intensity was detected as described above. Sirt5 concentration was adjusted to 15–30% substrate conversion. A negative control without enzyme and a positive control containing AMC instead of ZKsA were performed as well. Inhibition was calculated in % in relation to a DMSO control and was determined in triplicates.

#### Homogeneous ZMAL-Based Fluorescence Assay for HDAC1 and 6

The inhibition of HDAC1 and 6 by the S1th and PR-619 was determined via the ZMAL-assay according to the same general procedure described for sirtuins (2.2.1). 5 μL substrate (10.5 μM final assay concentration) were mixed with 3 μL of inhibitor in DMSO or DMSO and 10 μL of HDAC solution (concentration adjusted to 15–30% conversion) and filled up to 60 μL with assay buffer (50 mM Tris, 137 mM NaCl, 1 mM MgCl2, 2,7 mM KCl, 1 mg/ml BSA, pH = 8.0). The mixture was incubated at 37°C and 140 rpm for 1.5 h. For the stop solution Trichostatin A (3.3 μM) was used instead of nicotinamide. Fluorescence intensity was measured as described above. An enzyme free blank control and an AMC containing 100% control were performed as well and inhibition was calculated in % in relation to a DMSO control.

#### Jump Dilution Assay

To test the compounds for reversibility a jump dilution assay based on the trypsin-coupled ZMAL assay described above was used. In black 96 well plates (OptiPlateTM−96F, black, 96 well, Pinch bar design, PerkinElmer, USA) 1 μL Sirt1_134−747_ in assay buffer (50 mM Tris/HCl, 137 mM NaCl, 2.7 mM KCl, 1 mM MgCl_2_, Sirt1 concentration 100-fold higher than normally used) with 1 μL inhibitor in a concentration 10-fold higher than the IC_50_ were preincubated at room temperature for 10 min before the mixture was rapidly diluted 100-fold with assay mix [assay buffer, ZMAL (final assay concentration 500 μM), DMSO (final assay concentration 5% (v/v), NAD^+^ (final assay concentration 500 μM), pH 8.0] to a total volume of 200 μL. The reaction was stopped after 2.5, 5, 7.5, 10, 12.5, 15, 20, 25, and 30 min by addition of 60 μL stop solution [50 mM Tris, 100 mM NaCl, 6.7% (V/V) DMSO, trypsin 5.5 U/μL, 8 μM nicotinamide, pH 8.0]. The plate was again incubated at 37°C and 140 rpm for 20 min to get the free AMC from the deacetylated substrate by trypsin digest. Afterwards fluorescence intensity was measured in a microplate reader (λ_Ex_ = 390 nm, λ_Em_ = 460 nm, BMG POLARstar Optima, BMG Labtech, Germany). An enzyme-free blank control and a 100% conversion control containing AMC instead of ZMAL were performed additionally. Conversion was calculated in % in relation to the 100% control after subtraction of the blank fluorescence signal. Conversion in % was plotted against time with OriginPro 9G (OriginLab, USA) and an exponential fit was performed to fit the curves.

#### NAD^+^ Competition Assay

NAD^+^ competition was determined using the trypsin coupled ZMAL assay. Sirt1_134−747_, the ZMAL substrate and the inhibitors or DMSO were mixed and filled up to 55 μL in black 96-well plates as described above. The reaction was initiated by the addition of 5 μL of NAD^+^ in concentrations ranging from 62.5 to 2,000 μM final assay concentration. After 4 h of incubation at 37°C and 140 rpm the reaction was stopped by addition of the trypsin containing 60 μL stop solution, incubated again for 20 min at 37°C and 140 rpm and fluorescence was detected with a BMG POLARstar (λ_Ex_ = 390 nm, λ_Em_ = 460 nm, BMG POLARstar Optima, BMG Labtech, Germany). An enzyme-free blank control and a control containing AMC instead of ZMAL were measured additionally. Conversion was calculated in % in relation to the 100% control (AMC) after subtraction of the blank fluorescence signal. Conversion in % was plotted against NAD^+^ concentration with OriginPro 9G (OriginLab, USA) and an exponential fit was performed to fit the curves.

#### FOXO3a Substrate Competition Assay

Peptide substrate competition was measured using a homogeneous fluorescence-based fluorescamine assay similar to that previously reported as an activity assay for Sirt2 and Sirt3 with Ac-α-tubulin as a substrate (59). For Sirt1 a partial sequence of the physiological Sirt1 substrate FOXO3a [Ac-DSPSQLSK(Ac)WPPGTSS-NH2, custom synthesized by PSL, Heidelberg, Germany], hereafter called FOXO3a-ac, was used as substrate. Substrates were stored as 1 mM stocks in assay buffer [HEPES 25 mM, NaCl 137 mM, KCl 2.7 mM, MgCl_2_ 1 mM, Triton-X 100 0.015% (v/v), pH 8.0] and diluted with assay buffer to 240 μM FOXO3a-ac peptide (20 μM final assay concentration). In black 96-well plates (OptiPlateTM−96F, black, 96 well, Pinch bar design, PerkinElmer, USA) 0.05 μL Sirt1_134−747_ (3 mg/mL, final assay conversion 20–30%) was mixed with 5 μL of the diluted peptide and 3 μL of inhibitor dissolved in DMSO in various concentrations or DMSO as a control [final DMSO concentration 5% (v/v)] and filled up to 55 μL with assay buffer. After addition of 5 μL NAD^+^ (6 mM, final assay concentration 500 μM) to start the enzymatic reaction the plate was incubated for 1 h at 37°C and 140 rpm. Afterwards pH was adjusted with 5 μL of 0.1 M NaOH and the enzymatic reaction was stopped with stopping solution [8.73 mM nicotinamide (final assay concentration 4 mM), 0.455 mM fluorescamine (final assay concentration 62.5 μM) in acetone]. The fluorescence signal was measured using a microplate reader (λ_Ex_ = 390 nm, λ_Em_ = 485 nm, BMG POLARstar Optima, BMG Labtech, Germany). Additionally, a 100% inhibition control containing the physiological sirtuin inhibitor nicotinamide (6 mM final assay concentration) and a control simulating 100% conversion containing the deacetylated FOXO3a peptide (Ac-DSPSQLSKWPPGTSS-NH2, custom synthesized by PSL, Heidelberg, Germany) in equivalent concentration as the substrate were performed. Inhibition was calculated in % in relation to DMSO control after subtraction of the 100% inhibition fluorescence signal. IC_50_ values were determined using a non-linear regression to fit the dose-response curve with OriginPro 9G (OriginLab, USA).

#### Fluorescent Thermal Shift Assay (FTSA)

In a 96-well plate (Hard-Shell® PCR-plates, 96-well, thin-wall, BioRAD, USA) 14 μL Sirt1_134−747_ in assay buffer (0.3 mg/mL final assay concentration, 50 mM Tris/HCl, 137 mM NaCl, 2.7 mM KCl, 1 mM MgCL_2_, pH 8.0) were mixed with 1 μL inhibitor in DMSO (100 μM) or DMSO [final assay concentration 5% (v/v)] as a control and 5 μL SyproOrange (1:100 in assay buffer). The fluorescence intensity was monitored during a constant increase of temperature from 20 to 95°C, 1 K per 20 s, using a real-time-PCR-machine (C1000 TouchTM Thermal Cycler, CFX96TM Real-Time System, BioRAD, USA). Melting temperatures were calculated via GraphPad Prism according to a published procedure (60).

### Cellular Assays

#### DUB Labeling on HEK293T Cell Lysate

Pan-inhibition of deubiquitinases (DUBs) was tested using HEK293T cell lysate (human embryonic kidney cells, human). Cells were harvested and resuspended in HR buffer (50 mM Tris, 5 mM MgCl_2_, 250 mM sucrose, 1 mM DTT, pH 7.4). Cell lysis was achieved by sonication (Bioruptor, Diagenode, high intensity for 10 min with an ON/OFF cycle of 30 s) at 4°C and the cell debris was removed by centrifugation at 13,500 rpm for 15 min. Cell lysate protein concentration was determined with a NanoDrop spectrophotometer (NanoDrop One™ Spektrophotometer, Thermo Fisher, MA, USA) by measuring absorbance at 280 nm. Nineteen microliter of the lysate (2 μg/μL) were incubated with inhibitors dissolved in DMSO at various concentrations or DMSO as a control for 30 min at 37°C. Afterwards 1 μL of Rhodamine-Ub-PA (1 μM final assay concentration) was added to each sample, followed by further incubation for 40 min at 37°C (61). The labeling reaction was stopped by addition of NuPAGE 4× LDS sample buffer (Invitrogen Life Technologies, Carlsbad, CA, USA) containing β-mercaptoethanol and boiling for 7 min at 95°C. The proteins of the samples as well as protein marker (PageRuler™ Pre-stained Protein Ladder, 10–250 kDa, Thermo Fisher, MA, USA) were resolved by a 4–12% SDS-PAGE using the NuPAGE system with MOPS running buffer (Invitrogen Life Technologies, Carlsbad, CA, USA). The resulting gel was scanned with a Typhoon imager (GE Healthcare Life Sciences, USA) to visualize the Rhodamine-Ub-PA probe and Cy5 (marker). Subsequently the gel was stained with InstantBlue™ Protein Stain (Expedeon, UK) and scanned on a Amersham scanner (Amersham Typhoon gel and blot imaging system, GE Healthcare Life Sciences, USA).

#### γH2AX Level Determination

Sirt1 WT and KO MEFS cells were treated with 10 μM EX-527, 12 μM **S1th 13** (**6**), 20 μM **S1th 12** (**5**), 30 μM **S1th 10** (**7**) and 30 μM SirReal2 [Sirt2 inhibitor, synthesized according to (62)] for 48 h. Oxidative stress was induced using 10 μM Camptothecin 2 h. Whole-cell extracts were performed according to the Dignam protocol (63). Primary antibodies used for the western blot were anti-H2AX and anti-γH2AX (ab11175 and ab2893 resp., Abcam, UK). Densiometric analysis of the western blots was performed with Quantity One software (Bio-Rad Laboratories, Inc., USA).

#### Cell Proliferation Assay With HeLa or MCF7 Cells

1.5 × 10^4^ cells/well were seeded in 24-well plates and grown in DMEM 10% in presence of DMSO, 20 μM EX-527, 12 μM **S1th 13**, 20 μM **S1th 12**, or 30 μM **S1th 10**. The growth media containing the drugs was replaced every 48 h. Cells were collected and counted at the indicated times starting 24 h after seeding (0, 24, 48, 72, 96, and 120 h). The experiment was performed twice in duplicates.

#### Wound Healing Assay With HeLa Cells

For wound healing assay 6 × 10^5^ cells per well were seeded in 6-well plates and grown for 24 h in DMEM containing 10% FBS. After 24 h, a lineal gap between cells was created in the middle of the plate by scratching the cell monolayer with a 1 mL pipette tip in the same position in each well. Once the scratching is done, plates were washed twice with PBS and then grown in DMEM containing 1% FBS and the drugs in the same concentrations as for the proliferation assay. As described previously, the growth media 1% FBS containing the drugs was replaced every 48 h. Images were acquired in an Optimal microscope (Leica microscopes, DE) using Leica Application Suite X (LAS X) every 24 h (0, 24, 48, and 72 h) until the gap closed. The quantification of the area of the gap at the indicated times was performed with Image J-MRI Wound Healing Tool. The experiment was performed only once.

#### Colony Forming Assay With HeLa or MCF7 Cells

For colony assays, 50 cells per well were seeded in 6-well plates and grown in the same conditions with the same drug concentrations as for the proliferation assays. In this case, the growth media containing the drugs was replaced every 48 h. After 7–10 days, when isolated colonies were formed, wells were washed with PBS, and cells were fixed with cold methanol for 5 min at RT. Cells were stained with crystal violet for 10 min at RT and washed with H_2_O. Images were acquired using iBright (Thermo Fisher Scientific, MA, USA). For quantification, cells of each well were resuspended in 10% glacial acetic acid to dissolve crystal violet whose levels were monitored by measuring absorbance at 590 nm. The experiment was performed in duplicates.

#### Analysis of Acetylation Status in HeLa Cells by Western Blotting

5 × 10^4^ cells were plated in 6-well plates and grown with the conditions and drug concentrations as described for the proliferation assay. Cells were harvested at 48 h and resuspendend in protein loading buffer. After sonication the samples were centrifuged at 14,000 rpm in an Eppendorf microcentrifuge (Eppendorf, DE) and the supernatants were analyzed by western-blot. The following antibodies were used: histone H3 (Cell Signaling #9715), histone H3K9ac (Cell Signaling # C5B11), GAPDH (Cell Signaling #D16H11), p53 (ThermoFisher #PA527822), and p53K382ac (Abcam 75754). Quantification was carried out using imageJ.

### Computational Methods

3D structures of all compounds under study were generated from SMILES strings, and a subsequent energy minimization was carried out using the MMFF94x force field implemented in Molecular Operating Environment System (MOE) 2014.10 (Chemical Computing Group, Montreal, Canada). All compounds were used in their neutral form. A maximum of 100 conformations were generated for each ligand using the Conformational Search module implemented in MOE.

The structure of Sirt1 protein in complex with NAD^+^ and the small molecule inhibitor EX-527 was downloaded from the Protein Data Bank (PDB ID 4I5I) (64). In addition, crystal structures of Sirt2 in complex with the EX-527 analog CHIC35 and ADPR (PDB ID 5D7Q) and Sirt3 in complex with EX-527 and NAD^+^ (PDB ID 4BV3) were investigated. The protein structures were prepared by using the Structure Preparation module in MOE. Hydrogen atoms were added, for titratable amino acids the protonation state was calculated using the Protonate 3D module in MOE. Protein structures were energy minimized using the AMBER99 force field with a tethering force constant of (3/2) kT/2 (σ = 0.5 Å) for all atoms during the minimization (65). AM1-BCC charges were used for the studied ligands (66). All molecules except the zinc ion were removed from the structures.

Protein-ligand docking was performed using GOLD5.6 (67). For Sirt1, Ser442 was used to define the size of the grid box (15 Å radius). In case of Sirt2 and Sirt3, the corresponding Ser263 and Ser321 were used, respectively. Ten docking poses were calculated for each inhibitor. All other options were left at their default values. Using the docking setup, the cocrystallized inhibitors EX-527 and CHIC35 could be correctly docked with RMSD values below 0.6 Å. Virtual screening was carried out using program GOLD5.6 and the settings described above. To decrease calculation time, the Virtual Screening setup was used within GOLD5.6 and only the top-ranked pose was stored for further evaluation. In total 16 compounds were purchased and submitted to biochemical testing (eleven compounds from Princeton Biomolecular Research, one from Sigma-Aldrich and four from Enamine, Supplementary Table 1). All docking scores displayed in Supplementary Table 1.

## Results

### Docking

**Figure 2:**
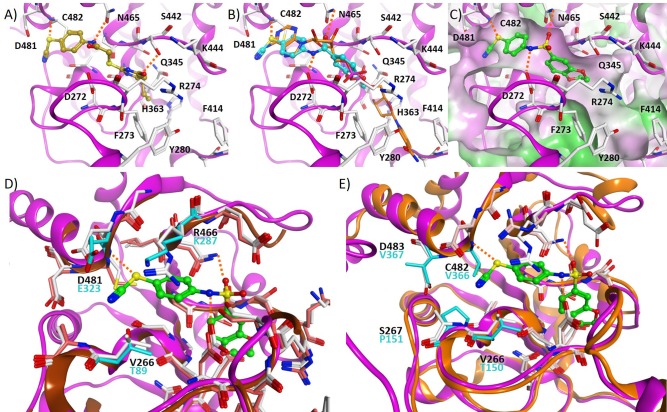
Sirt1 ribbon is colored purple, hydrogen bonds are shown as dashed lines. **(A)** Docking pose obtained for **S1th 1** (colored beige) at the Sirt1 NAD^+^ binding pocket. **(B)** Docking pose obtained for **S1th 7** (colored cyan) at the Sirt1 NAD^+^ binding pocket, NAD^+^ (colored orange) is shown for comparison. **(C)** Docking pose obtained for **S1th 13** (colored green) at the Sirt1 NAD^+^ binding pocket. The molecular surface of the binding pocket is colored according to the hydrophobicity (hydrophobic = green, hydrophilic = magenta). **(D)** Superimposition of the crystal structure of Sirt1 (PDB ID 4I5I, magenta colored ribbon, white colored residues) with docked **S1th 13** (colored green) and the crystal structure of Sirt2 (PDB ID 5D7Q, brown colored ribbon, pink colored residues). The amino acid residues of Sirt2 that are different are colored cyan. **(E)** Superimposition of the crystal structure of Sirt1 (PDB ID 4I5I, magenta colored ribbon, white colored residues) with docked **S1th 13** (colored green) and the crystal structure of Sirt3 (PDB ID 4BV3, orange colored ribbon, salmon colored residues). The amino acid residues of Sirt3 that are different are colored cyan.

**Figure 3:**
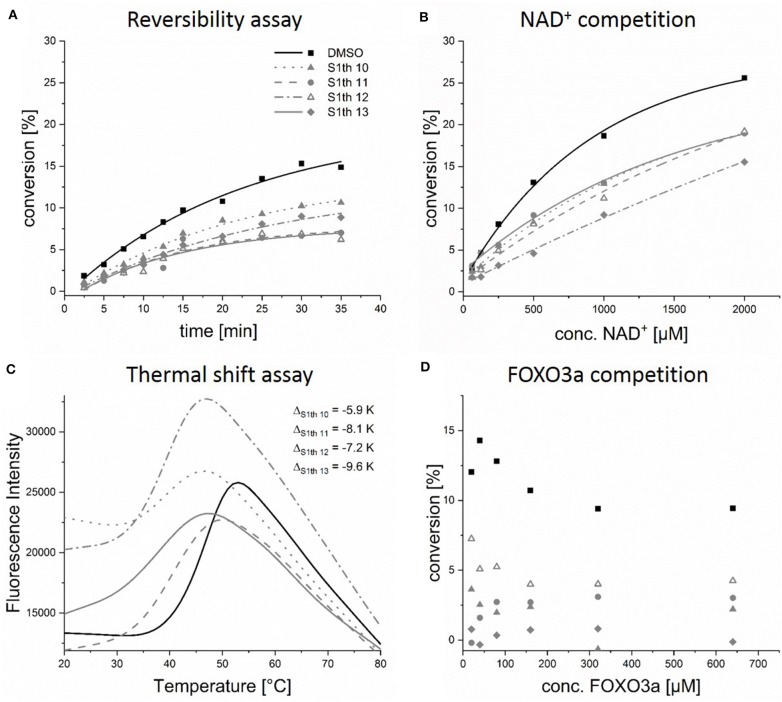
*In vitro* characterization of the thiocyanates. Evaluation of reversibility, competition with NAD^+^ or the peptide substrate FOXO3a and enzyme stabilization; **(A)** Sirt1 and the respective thiocyanate inhibitor (50 μM) were preincubated for 10 min and then rapidly diluted 1:100 with assay solution (buffer, ZMAL, NAD^+^), activity of Sirt1 was measured at different time points. Increasing activity can be observed for non-covalent inhibitors; **(B)** thiocyanate inhibitors (100 μM) were incubated with increasing concentrations of NAD^+^, showing that the inhibitors can be replaced by NAD^+^; **(C)** binding of **S1th 10–13** (100 μM) to Sirt1 leads to a strong decrease of *T*_*m*_; **(D)** S1th (100 μM) were incubated with increasing concentrations of the peptide substrate FOXO3a, the inhibitors are not competitive toward FOXO3a. All biochemical assays were performed at least twice in duplicates.

Based on a previously collected library of putative sirtuin inhibitors we carried out a virtual screening using the GOLD5.6 docking software and the available crystal structure of Sirt1 in complex with the inhibitor EX-527 and NAD^+^ (64). A first *in vitro* screening (for primary *in vitro* screening data see Supplementary Table 2) on the three class I sirtuins indicated a thienopyrimidone carrying a thiocyanate moiety which we termed **S1th 1** (**2**) (structure Figure 1) as a promising hit for Sirt1 inhibition. Two structural analogs of **S1th 1** with the same heterocyclic system but different functional groups were docked to the NAD^+^ binding site of Sirt1 and were submitted to a second round of biochemical testing (Table 1). Among the three selected thienopyrimidones only **S1th 1** was able to inhibit Sirt1 in the micro molar range (IC_50_ 13 μM). The docking pose of the active analog showed that the phenyl thiocyanate moiety is located in the adenine pocket, engaging in a hydrogen bond to Cys482 (backbone NH, Figure 2A). The pyrimidine ring is located in the polar phosphate pocket of Sirt1 and shows a hydrogen bond to Ser442. A third hydrogen bond was observed between the amide group of the inhibitor and Gln445. The two inactive analogs (**OSSK_338451** and **OSSK_531963**, structures Supplementary Figure 1) showed a similar binding mode lacking an interaction in the adenine pocket. Due to the close proximity of the potentially reactive thiocyanate group of **S1th 1** and Cys482 we speculated that an irreversible binding might occur. Therefore, we tested the reversibility of enzyme inhibition as well as NAD^+^ competition and competition toward a peptide analog of the physiological Sirt1 substrate FOXO3a to confirm the proposed binding mode. *In vitro* results showed that the inhibitors are reversible binders and NAD^+^ competitive but not substrate competitive which is in agreement with the predicted binding mode of the thiocyanates (Figure 3).

**Table 1:** Inhibition of class I sirtuins by **S1th 1** and its two structural analogs **OSSK_338451** and **OSSK_531963** (2nd round of *in vitro* screening).

**Compound**	**IC**_**50**_ **[μM] or % inhibition @ 50 μM**		
	**Sirt1**	**Sirt2**	**Sirt3**
S1th 1	13 ± 0.6	23%	n.i.^*^
OSSK_33845	n.i.	n.i.	n.i.
OSSK_531963	n.i.	n.i.	n.i.

* *n.i., no inhibition (<10%)*.

To confirm the importance of the thiocyanate group we screened the whole Princeton BioMol. Res. Compound collection virtually (considering only phenyl thiocyanates) and docked the resulting 113 thiocyanates to Sirt1. Eight promising hits were cherry picked, purchased and submitted to a third round of biochemical testing (Table 2). Among the eight compounds, **S1th 7** (**3**) showed increased inhibition compared to **S1th 1** with an IC_50_ of 6.34 μM (Table 2) which is also supported by the best docking score. The predicted binding pose of **S1th 7** shows two hydrogen bonds to Asp272 and Asn465 (Figure 2B). Since we encountered solubility problems of the active hits in cellular testing at higher concentrations, we purchased six more polar compounds and submitted them to a fourth round of biochemical testing. **S1th 11** (**4**), **12** (**5**), and **13** (**6**) (from Enamine) were found to be better soluble and equally active as **S1th 7**. The binding mode of the active hit **S1th 13** (IC_50_ of 5.2 μM, Table 3) is similar to that observed for the previous hits showing interactions with Cys482, Asn465 and in addition to Asp272 (Figure 2C).

**Table 2:** Inhibition of class I sirtuins by thiocyanate analogs of 3rd round of virtual screening (**S1th 2–9**).

**Compound**	**IC**_****50****_ **[μM] or % inhibition @ 50 μM**		
	**Sirt1**	**Sirt2**	**Sirt3**
S1th 2	6.4 ± 0.7	n.i.^*^	n.i.
S1th 3	2.8 ± 0.5	n.i.	n.i.
S1th 4	5.3 ± 0.5	n.i.	n.i.
S1th 5	9.4 ± 1.4	n.i.	n.i.
S1th 6	5.1 ± 0.6	n.i.	n.i.
S1th 7	6.3 ± 0.5	n.i.	n.i.
S1th 8	40%	n.i.	n.i.
S1th 9	n.i.	n.i.	n.i.

* *n.i., no inhibition (<10%)*.

**Table 3:** Inhibition of sirtuins 1, 2 3 and 5 by thiocyanate analgues of 4th round of virtual screening **(S1th 10-13)** and **PR-619**.

**Compound**	**IC**_****50****_ **[μM] or % inhibition @ 50 μM**			
	**Sirt1**	**Sirt2**	**Sirt3**	Sirt5^**^
S1th 10	23 ± 6.0	n.i.^*^	n.i.	n.i.
S1th 11	6.3 ± 0.8	n.i.	n.i.	n.i.
S1th 12	5.9 ± 1.4	n.i.	n.i.	n.i.
S1th 13	5.2 ± 1.0	n.i.	n.i.	n.i.
PR-619	2.7 ± 0.2	36%	n.i.	28%

* *n.i., no inhibition (<10%)*,

** *inhibition tested @ 10 μM*.

All active inhibitors, both from the 3rd and the 4th round, retained the extremely high selectivity of the initial hit for Sirt1 over the isotypes Sirt2 and 3. Docking to Sirt2 and Sirt3 was subsequently carried out for the active hits in order to rationalize the observed selectivity. In case of Sirt2, there are three different amino acid residues in the putative thiocyanate binding pocket that affect the docking results (Figure 2D): Val266, Arg466, and Asp481 of Sirt1 are substituted by Thr89, Lys287, and Glu323 in the Sirt2 structure, respectively. The inhibitors could be docked in a similar orientation to the Sirt2 binding pocket, however with less favorable docking scores. Especially the interaction of the thiocyanate phenyl ring with Arg466 is lost in case of Sirt2, which might explain the lower docking scores. In case of Sirt3, there are four amino acid residues substituted in the putative binding pocket. Val266, Ser267, Cys482, and Asp483 of Sirt1 are substituted by Thr150, Pro151, Val366, and Val367 in the Sirt3 structure (Figure 2E). Val366, Val367, and Pro151 are restricting the size of the putative thiocyanate binding pocket and consequently the thiocyanate moiety is not able to interact with the protein as observed for Sirt1.

### *In vitro* Characterization of S1th

We wanted to further elucidate the binding mode and selectivity of the S1th. Apart from the closely related class I sirtuins (Sirt2 and 3) also inhibition of Sirt5 as a representative of other sirtuin classes was tested. Although Sirt5 sequence and structure in general shows less overlap with Sirt1 than the class I sirtuins, the active side residue Cys482 which is explicitly important for S1th binding is conserved in Sirt5. Yet, no inhibition of Sirt5 by **S1th 10–13** at 10 μM was observed. In general, thiocyanates are known to act as chelating groups making them candidates for inhibition of ion-dependent enzymes like classical HDACs which feature a zinc ion in their active site. It has been shown that cruciferous vegetable isothiocyanates like sulforaphane can act as potent pan-HDAC inhibitors (68). Consequently, to further investigate the selectivity of our new thiocyanate Sirt1 inhibitors, they were tested against two representative zinc dependent HDACs, HDAC1 (Class I) and HDAC6 (Class II). HDAC1 was not inhibited by the tested thiocyanates at 100 μM at all. For HDAC6 a very weak inhibition by **S1th 10** (**7**), **12** and **13** (19, 36, and 29% at 100 μM) was observed (Table 4). Another likely off-target effect of thiocyanates could be the inhibition of deubiquitinases (DUBs). Deubiquitination activity of these enzymes relies on a cysteine residue in the catalytic core which acts as a nucleophile (69). Most known DUB inhibitors therefore feature a functional group that can form specific interactions with this cysteine. PR-619 (**8**), a compound featuring two aromatic thiocyanate moieties, was shown to inhibit more than twenty DUBs with IC_50_ values ranging from 5 to 20 μM and, as we could show, is also able to inhibit Sirt1 with an IC_50_ of 2.7 ± 0.2 μM (70, 71). HDAC1 and 6 are inhibited to ~50% residual activity by PR-619 as well. The S1th however did not show inhibition of DUBs which we could show in a fluorescence based activity assay using HEK-293 cell lysate and a Rhodamine-Ub-PA probe (Supplementary Figure 2). These results support the excellent selectivity of the S1th not only amongst sirtuins but also over other possible off-targets.

**Table 4:** Selectivity over histone deacetylases and deubiquitinases compared to pan-DUB inhibitor PR-619.

**Compound**	**% inhibition @ 100 μM**		**Qualitative pan-DUB inhibition**	
	**HDAC1**	**HDAC6**	**@ 10 μM**	**@ 50 μM**
S1th 10	n.i.^*^	29%	n.i.	n.i.
S1th 11	n.i.	n.i.	n.i.	n.i.
S1th 12	n.i.	36%	n.i.	n.i.
S1th 13	n.i.	19%	n.i.	n.i.
PR-619	54%	49%	+	+++

* *n.i., no inhibition (<10%), + low inhibition, +++ full inhibition*.

To better understand the binding of the S1th a series of fluorescent thermal shift assay (FTSA) experiments was performed. Binding of a ligand usually results in a stabilization of the protein which can be observed as an increase of the melting temperature (*T*_*m*_) in FTSA. For example, binding of the NAD^+^ metabolite adenosine diphosphate ribose (ADPR), which is formed during the catalytic reaction of the sirtuins, leads to a stabilization up to 4 K. The known Sirt1 binders EX-527 and SRT1720 (72) only cause a very small shift or no shift at all (Supplementary Figure 3a). For the S1th however we observed a strong left shift of the *T*_*m*_ from 5.9 up to 9.6 K for the most potent inhibitors of the fourth round at 100 μM (Figure 3). The decrease of *T*_*m*_ is concentration dependent for all tested compounds (**S1th 10–13**), resulting in a smaller ΔT at lower concentrations (Supplementary Figure 3c). Shifts of *T*_*m*_ to decreased temperatures have been associated with an apparent destabilization of the protein by covalent binding compounds, detergent-like compounds or compounds that extract stabilizing ions from the structure in several cases (73). This could be a hint toward a covalent interaction of the thiocyanate moiety with Cys482. However, another widely recognized explanation for left shifts is that these compounds bind more strongly to a conformation different from the native one (74, 75). In regard to this, one has to take into account that *T*_*m*_ is considerably more affected by entropy than by enthalpy. Consequently, enthalpy-driven binding to the native state can be outnumbered by weaker entropy-driven binding to a different conformation or even the denatured state resulting in a left shift (76). The shift of *T*_*m*_ induced by **S1th 10–13** can be reversed through addition of ADPR. As the thiocyanates are binding to the NAD^+^ pocket they are also competitive toward the physiological NAD^+^ metabolite ADPR. Simultaneous application of ADPR and the S1th resulted in a significantly smaller decrease of *T*_*m*_(Sirt1) than treatment with S1th alone (Supplementary Figure 3b). To ensure that the observation of a left shift is specific for Sirt1 the compounds were also tested in an FTSA using Sirt2. There was no decrease of *T*_*m*_ observed, in fact binding of the thiocyanates leads to a very small positive shift (0.2–0.5 K) of *T*_*m*_ for Sirt2 (Supplementary Figure 3d). As the Cys482 residue is conserved in five out of the seven human sirtuin isotypes including Sirt2 and 5, this data shows a unique and specific binding mode of the thiocyanates to the Sirt1 structure that was already proposed by the docking to Sirt1, 2 and 3 (77).

### Effects of S1th in Cellular Systems

#### Sirt1 Dependent Effects in Mice Embryonic Fibroblasts (MEFs)

**Figure 4:**
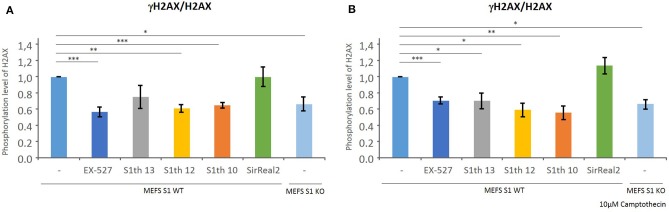
Quantification of γH2AX/H2AX levels in MEF cells after Sirt1 depletion or incubation with various inhibitors by western blot analysis (original western blots: Supplementary Figure 4) with anti-γH2AX and anit-H2AX. γH2AX/H2AX levels are decreased in Sirt1 KO cells and upon addition of EX-527 (10 μM), **S1th 10** (30 μM), **S1th 12** (20 μM), or **S1th 13** (12 μM). The selective Sirt2 inhibitor SirReal2 (30 μM) does not affect γH2AX/H2AX levels. Addition of Camptothecin increases DNA damage in cells **(A)** γH2AX/H2AX levels without Camptothecin; **(B)** γH2AX/H2AX levels after addition of Camptothecin (10 μM). The experiments were performed at least three times each. *p < 0.05, **p < 0.01, ***p < 0.001.

As described previously by Wang et al., the phosphorylation level of H2AX in mice embryonic fibroblasts (MEFs) is decreased if Sirt1 is depleted or inhibited (42). Phosphorylation of H2AX is a key step in DNA damage sensing. Determination of γH2AX/H2AX levels in MEFs via western blotting was employed to show target engagement in cells of our new class of Sirt1 inhibitors. To demonstrate the maximum change in phosphorylation possible, the γH2AX/H2AX levels of wild type MEFs and Sirt1 KO MEFs were determined. The effect of **S1th 10**, **12**, and **13** (30, 20, and 12 μM, respectively) was compared to that of EX-527 (10 μM) as a positive control and to the specific Sirt2 inhibitor SirReal2 (30 μM) as a negative control (Figure 4, for western blots see Supplementary Figure 4). All three inhibitors showed a reduction of γH2AX/H2AX levels and the observed effect is similar to that of EX-527. The effect of the sirtuin 1 inhibitors becomes even more apparent when the topoisomerase I inhibitor Camptothecin is added to the cells (78). Treatment with Camptothecin induces DNA damage which in functional cells leads to elevated phosphorylation levels of H2AX. When EX-527 or the S1th were administered to these cells again a strong decrease of γH2AX/H2AX was detected. This shows that in cells with higher stress levels through DNA damage, the S1th can significantly alter DDR.

#### Impact of S1th on Cervical Cancer Cells (HeLa)

**Figure 5:**
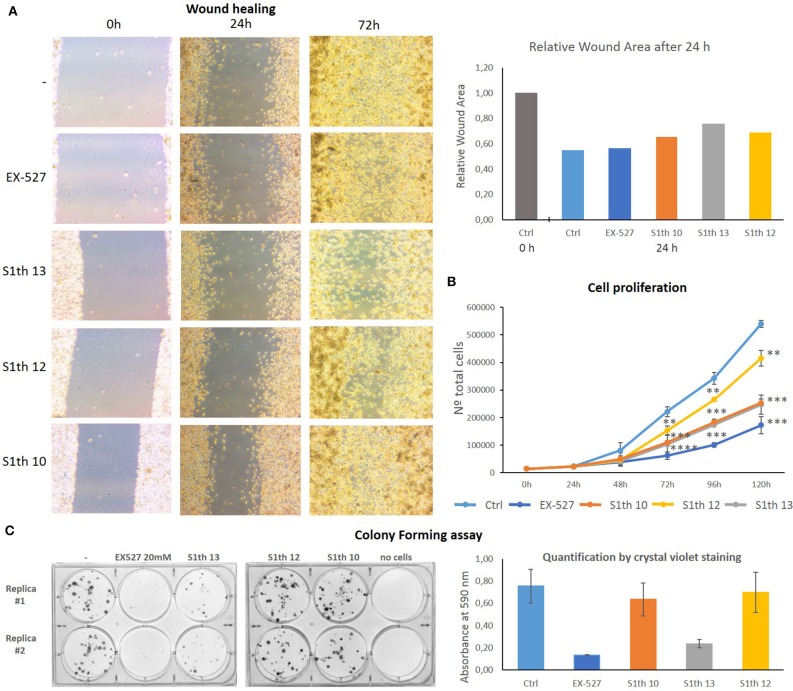
**(A)** Impact of EX-527 and **S1th 10**, **12**, and **13** on cell migration of HeLa cells in a wound healing assay. All compounds were able to impair cell migration. The S1th showed a clear effect already after 24 h, while a decrease of migration only came apparent after 72 h for the positive control EX-527. This experiment was only carried out once. **(B)** Impact of **S1th 10**, **12**, and **13** and EX-527 on cell proliferation of HeLa cells. All compounds are able to slow down cell proliferation of HeLa cells, with **S1th 12** only showing a mild effect. The experiments were performed at least three times each. *p < 0.05, **p < 0.01, ***p < 0.001, ****p < 0.0001. **(C)** Impact of EX-527 and **S1th 10**, **12**, and **13** on colony forming capabilities of HeLa cells. Colony forming is significantly decreased by EX-527 and **S1th 13**, but barely affected by **S1th 12** and **10**. Experiments were carried out in duplicates and quantification was done by staining the colonies with crystal violet and measuring absorbance at 590 nm.

**Figure 6:**
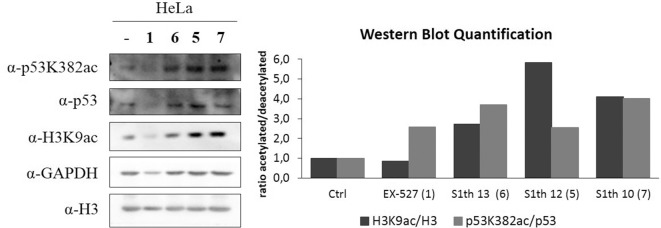
Western blot analysis of acetylation status of p53K382 and H3K9 in HeLa cells. A α-GAPDH antibody was used as a loading control. Histone H3 and p53 expression was monitored by α-H3 and α-p53 antibodies and was not altered by application of the inhibitors, while α-H3K9ac and α-p53K382ac indicate the impact on acetylation status upon inhibitor treatment. **1** (EX-527) was used as a positive control, however it did not show clear hyperacetylation of H3K9. In contrast, a clear effect on acetylation of these Sirt1 targets is observed when **5**, **6**, and **7** (**S1th 12**, **13**, **10** resp.) are applied. Quantification of the shown blot is displayed as the ratio between acetylated and deacetylated protein in relation to a control where no inhibitor was applied.

After showing Sirt1 dependent effects in non-cancerous mouse cells we wanted to investigate whether our new inhibitors have an impact on proliferation, migration and colony forming properties of human cancer cells. As already mentioned Sirt1 can play very contradictory roles in different tissues and even within one cancer type. Still, cases have been reported where Sirt1 inhibition impairs cell growth. For cervical cancer cells (HeLa) effects on cell proliferation upon administration of EX-527 have been reported (79). Based on these findings we chose HeLa cells for further examination of the S1th. The three best characterized inhibitors **S1th 10**, **12**, and **13** as well as EX-527 were administered to HeLa cells and the effects on cell proliferation, migration and colony forming were observed (Figure 5). EX-527, **S1th 10** and **S1th 13** significantly decreased cell proliferation, with EX-527 being slightly more effective than the S1th. **S1th12** only showed a mild effect on proliferation. Interestingly, even though having the most impact on cell proliferation, EX-527 failed to impair migration of HeLa cells in a wound healing assay within the first 24 h. A monolayer of HeLa cells was plated and a “wound” was introduced by scratching. Through cell migration the cells grow back together to heal the wound in the monolayer. Other than EX-527, the S1th all showed good inhibition of cell migration already after 24 h. **S1th 13** appeared to be most effective showing an ~50% lower reduction of total wound area compared to a control where no drug was applied. After 72 h also EX-527 showed a mild effect on wound healing and the inhibition by the S1th that could already be observed after 24 h became more apparent. In consistency with the results on proliferation and migration also a colony forming assay proved **S1th 13** to be the most potent drug of our new class. EX-527 completely suppressed colony forming of HeLa cells. **S1th 13** proved to be almost as potent as EX-527 while for **S1th 10** and **12** only mild effects were observed. Effects of the S1th on proliferation and colony forming were also confirmed in initial studies in the breast cancer cell line MCF7 (Supplementary Figure 5). In addition, we performed western blot analysis of the Sirt1 substrates H3K9ac and p53K382ac (Figure 6). To determine the acetylation status binding of α-H3K9ac and α-p53K382ac were compared to α-H3 and α-p53 binding, respectively. Thereby stable expression of H3 and p53 in all samples was ensured and additionally α-GAPDH was used as a loading control. All three S1th tested were able to significantly increase H3K9 and p53K382 acetylation and thereby affirm selective Sirt1 inhibition in HeLa cells. For EX-527 no hyperacetylation of H3K9 but increased acetylation levels of p53K382 were observed.

## Discussion

To further investigate the role of Sirt1 in cancer, new selective inhibitors for this isotype will be of great value. However, so far only few such inhibitors have been reported. In this study we identified a new class of selective and potent Sirt1 inhibitors, the Sirtuin 1 inhibiting Thiocyanates (S1th) by an iterative process of virtual screening and biochemical testing. Molecular docking of the S1th to the crystal structure of Sirt1 in complex with its cofactor NAD^+^ revealed their putative binding mode. In general, the inhibitors are proposed to bind to the NAD^+^ binding pocket of Sirt1. This could be confirmed in a competition assay, showing competition between the inhibitors and the cofactor NAD^+^ but not toward a peptide substrate analog. The most potent inhibitor of this class **S1th 13** is thought to engage in two hydrogen bonds with Asn465 and Asp272 and a potentially covalent interaction with Cys482. As indicated by competition assays binding of the S1th is reversible leading to the conclusion that even though the interaction with Cys482 could be covalent it is also fully reversible. Fast reversible covalent inhibitors have been reported before e.g., for kinases (80). In thermal shift assays covalent inhibitors often show a characteristic left shift, as they can destabilize the thermodynamically most stable conformation of an enzyme or can stabilize a different confirmation. S1th binding resulted in a strong left shift of Sirt1 melting temperature. Interestingly, the Sirt2 melting temperature was not affected at all by the S1th, although the respective cysteine residue is conserved in Sirt2 (Cys324). These results indicate that no unspecific binding of the S1th's thiocyanate moiety occurs and the interaction between the thiocyanate and Cys482 in Sirt1 is highly selective. Further we could demonstrate that S1th are selective over sirtuin isotypes 3 and 5, representatives of HDAC class I and II (HDAC 1 and 6) as well as a set of deubiquitinases (DUBs). Selectivity over DUBs is especially remarkable since they are known to be inhibited by thiocyanates through binding of a catalytically relevant cysteine residue in the active site of DUBs. The thiocyanate PR-619 for example is a pan-DUB inhibitor and as we showed also inhibits Sirt1 with a low micro molar IC_50_. After ensuring high selectivity and potency of our new inhibitor class we wanted to prove target engagement in cells. **S1th 10**, **12**, and **13** were applied to MEF cells and the effect on H2AX phosphorylation was detected. H2AX is a DNA damage sensor that gets phosphorylated upon DNA damage. Phosphorylation of H2AX was significantly decreased by **S1th 10**, **12**, and **13** as well as by the positive control EX-527 while a selective Sirt2 inhibitor (SirReal2) did not show any effect. The reduction of γH2AX/H2AX levels observed after application of Sirt1 inhibitors was similar as in Sirt1 KO MEFs. Additional treatment with camptothecin, a drug that induces DNA damage through inhibition of topoisomerase I and thereby increases γH2AX/H2AX levels, didn't surpass the inhibitory effect of the S1th or the positive control but even seemed to increase the efficacy especially for **S1th 13**. Finally, we tested the effects of S1th on proliferation, migration and colony forming capabilities of human cervical cancer cells. The cervical cancer cell line HeLa was treated with **S1th 10**, **12**, **13** and EX-527 as a positive control. **S1th 13** showed significant inhibition of proliferation, migration and colony forming while **S1th 10** and **12** only had moderate effects. EX-527 also showed robust inhibition of cell proliferation and colony forming, however for migration a clear effect became apparent only 72 h after treatment but not already after 24 h, as observed for the S1th. Finally, western blot analysis confirmed that the effect of S1th in HeLa cells is associated with concomitant hyperacetylation of H3K9 and p53K382.

Although the *in vitro* potency of the S1th is yet lower than that of the reference EX-527, their discovery, especially their very high selectivity, still opens up new possibilities. Remarkably, unlike EX-527 that has strongly decreased potency in the cellular setting as compared to the biochemical assay, they show similar potencies in cells as *in vitro*. This demonstrates their high potential to further study the role of Sirt1 in cellular model systems for cancer research but also in other diseases. The structural knowledge obtained from available crystal structures of Sirt1 and our docking studies can be utilized for future inhibitor optimization. Knowing that thiocyanates are able to engage in a specific interaction right in the catalytic core of the enzyme, new structures can be designed and synthesized. Already now the S1th present a valid alternative to EX-527 for cellular studies.

## Data Availability Statement

All datasets generated for this study are included in the article/Supplementary Material.

## Author Contributions

MJ and WS contributed conception and design of the study. ZA performed the computational studies. HO and JGa performed selectivity testing on DUBs. KS performed selectivity testing on HDACs. SS performed initial *in vitro* testing. Cellular tests were planned by AV and JGo and performed by JGo. OE and LZ designed and executed expression and purification of enzymes for *in vitro* testing. NW performed *in vitro* studies and wrote major parts of the manuscript. MJ, ZA, and WS wrote sections of the manuscript. All authors contributed to manuscript revision, read and approved the submitted version.

## Conflict of Interest

The authors declare that the research was conducted in the absence of any commercial or financial relationships that could be construed as a potential conflict of interest.
