# Supplementary material for: Sirtuin 1 Inhibiting Thiocyanates (S1th)—A New Class of Isotype Selective Inhibitors of NAD+ Dependent Lysine Deacetylases
Source: Front Oncol. 2020 Apr 30;10:657. doi: 10.3389/fonc.2020.00657 (PMC7203344; doi:10.3389/fonc.2020.00657)
Supplement: Supplementary file 1 [file Table_1.docx]

Supplementary Material

**Supplementary Table 1.** Docking scores, calculated logP/logS values and commercial names and sources for studied thiocyanate derivatives.

| **Compound** | **name** | **vendor** | **logP (o/w)** | **logS** | **Gold-score**  **Sirt1** | **Gold-score**  **Sirt2** | **Gold-score**  **Sirt3** | **Test round** |
| --- | --- | --- | --- | --- | --- | --- | --- | --- |
| **S1th 1** | OSSK_221646 | Princeton BioMol. Res. | 2.88 | -6.21 | 75.0 | 75.0 | 69.9 | 1/2 |
|  | OSSK_338451 | Princeton BioMol. Res. | 2.90 | -5.11 | 62.83 | 79.6 | 68.9 | 2 |
|  | OSSK_531963 | Princeton BioMol. Res. | 1.42 | -4.53 | 69.3 | 76.8 | 75.0 | 2 |
| **S1th 2** | OSSK_715368 | Princeton BioMol. Res. | 4.18 | -7.14 | 84.3 | 78.4 | 79.9 | 3 |
| **S1th 3** | OSSK_999417 | Princeton BioMol. Res. | 2.77 | -6.81 | 93.2 | 84.9 | 81.1 | 3 |
| **S1th 4** | OSSK_702126 | Princeton BioMol. Res. | 3.37 | -7.09 | 82.7 | 75.2 | 74.9 | 3 |
| **S1th 5** | OSSL_081720 | Princeton BioMol. Res. | 1.41 | -5.06 | 79.5 | 74.3 | 73.8 | 3 |
| **S1th 6** | OSSK_702117 | Princeton BioMol. Res. | 3.31 | -6.88 | 86.1 | 75.6 | 76.7 | 3 |
| **S1th 7** | OSSK_629100 | Princeton BioMol. Res. | 2.73 | -5.06 | 74.9 | 68.5 | 71.4 | 3 |
| **S1th 8** | OSSL_301409 | Princeton BioMol. Res. | 1.83 | -2.64 | 59.3 | 58.7 | 51.0 | 3 |
| **S1th 9** | OSSK_991515 | Princeton BioMol. Res. | 3.00 | -4.98 | 70.1 | 67.5 | 61.2 | 3 |
| **S1th 10** | Z169886730 | Enamine | 1.21 | -2.97 | 66.3 | 49.4 | 58.3 | 4 |
| **S1th 11** | Z44591052 | Enamine | 2.52 | -3.93 | 75.4 | 80.1 | 66.5 | 4 |
| **S1th 12** | Z1556189872 | Enamine | 0.36 | -3.53 | 80.2 | 71.4 | 78.0 | 4 |
| **S1th 13** | Z45638535 | Enamine | 2.53 | -4.75 | 83.4 | 74.4 | 71.0 | 4 |
|  | PR-619 | Sigma-Aldrich | 0.73 | -3.10 | 60.1 | 60.8 | 59.5 | 4 |
|  | EX-527 | Selleckchem |  |  |  |  |  |  |

**Supplementary Figure 1.** Structures of second and third round docking suggestions**.**


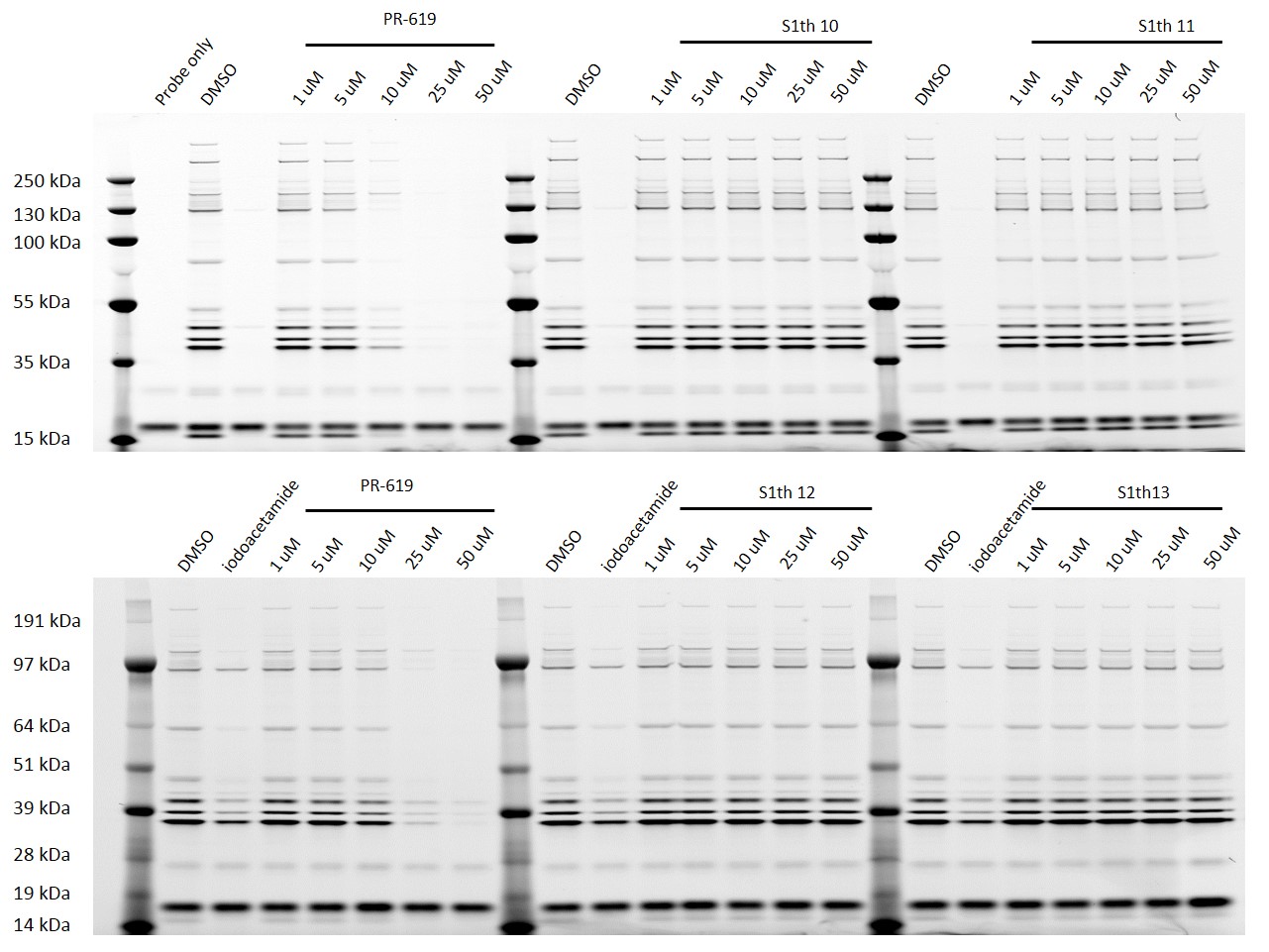
**Supplementary Figure 2.** Selectivity of the thiocyanates over DUBs. The thiocyanate DUB inhibitor PR-619 and iodoacetamide were used as positive controls. HEK cell lysate was incubated with a Rhodamine labeled activity-based probe for deubiquitinases to illustrate DUB activity. Fluorescence of Rhodamine probe was measured. Visible bands show active DUBs, while bands disappear when a respective DUB is inhibited. A) Comparison of PR-619 to **S1th10** and **11**; B) Comparison of PR-619 to **S1th12** and **13**; PR-619 inhibits all DUBs tested in a concentration dependent manner but no effect on DUB activity by the thiocyanates was observed.

**
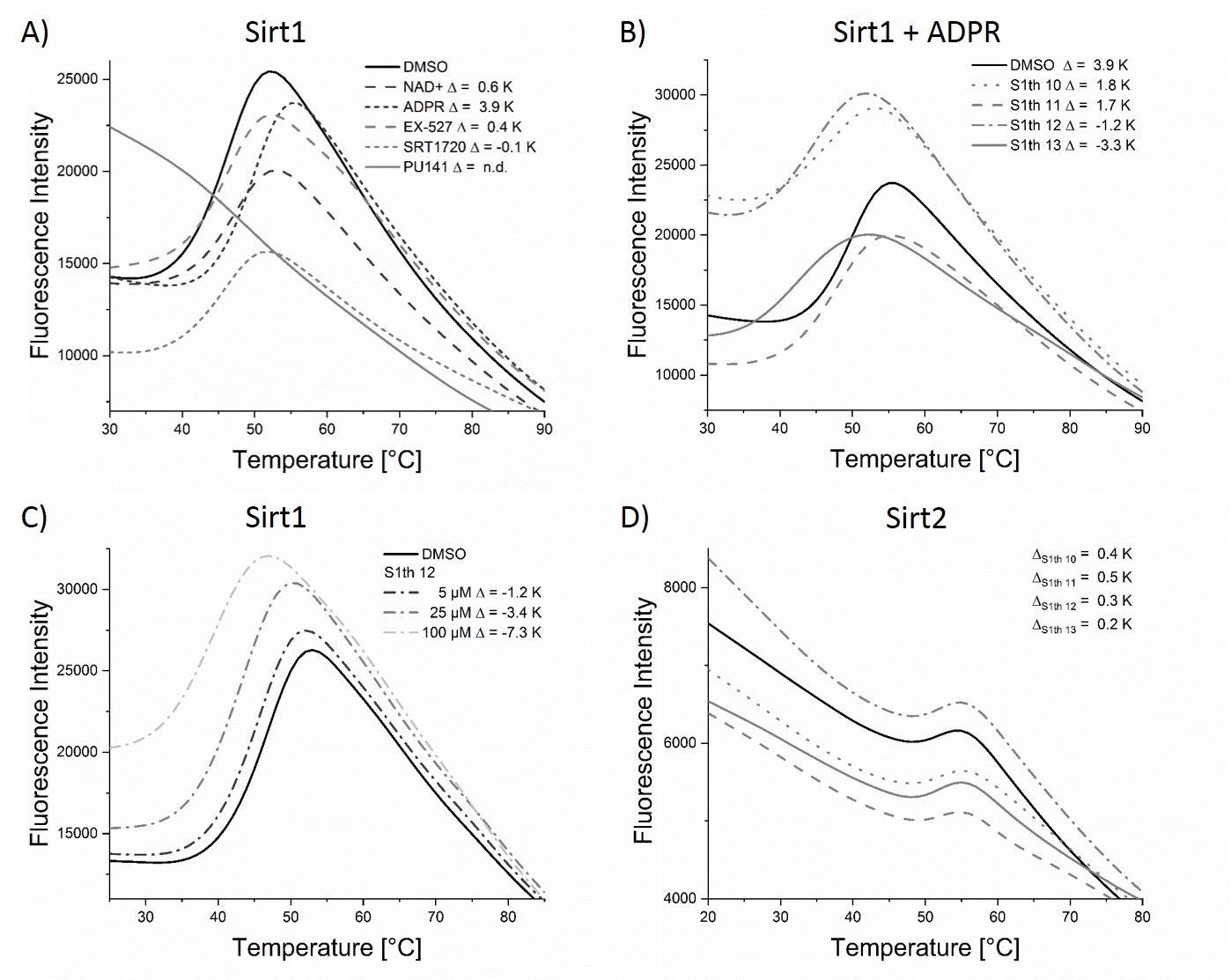
**

**Supplementary Figure 3.** FTSA analysis of various compounds with Sirt1 and 2. A) *T_m_* of Sirt1 is barely effected by binding of EX-527 or SRT1720, but slightly increased when NAD^+^ is added and strongly increased when ADPR is added, no reasonable melting curve is obtained with the unselective thiol reactive isothiazolone PU141, possibly due to unspecific interactions with the enzyme or the dye; B) addition of ADPR significantly decreases destabilizing effect of thiocyanates on Sirt1, due to competition between ADPR and the S1th; C) negative shift of the melting curve upon binding of the S1th is concentration dependent, here shown representatively for **S1th 12**; D) binding of the thiocyanates to Sirt2 leads to a small increase of Sirt2 melting temperature. All FTSA experiments were carried out at least twice in duplicates.


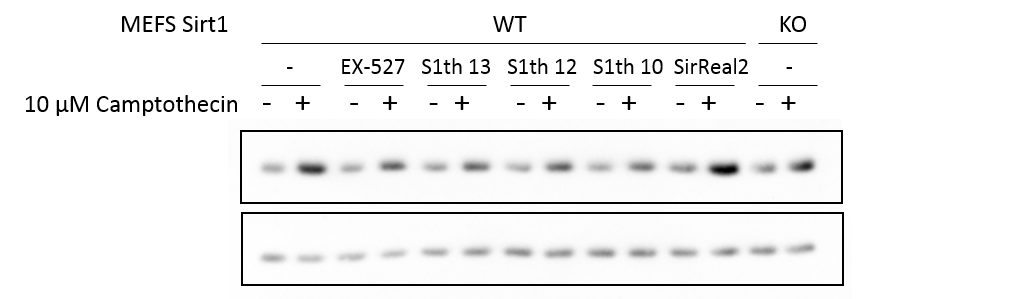


**Supplementary Figure 4.** Western blot analysis of γH2AX/H2AX levels in MEF cells treated with various sirtuin inhibitors compared to Sirt1 KO cells using anti-γH2AX and anti-H2AX; w and w/o camptothecin addition; EX-527 and the thiocyanates lead to a strong decrease of γH2AX/H2AX levels.


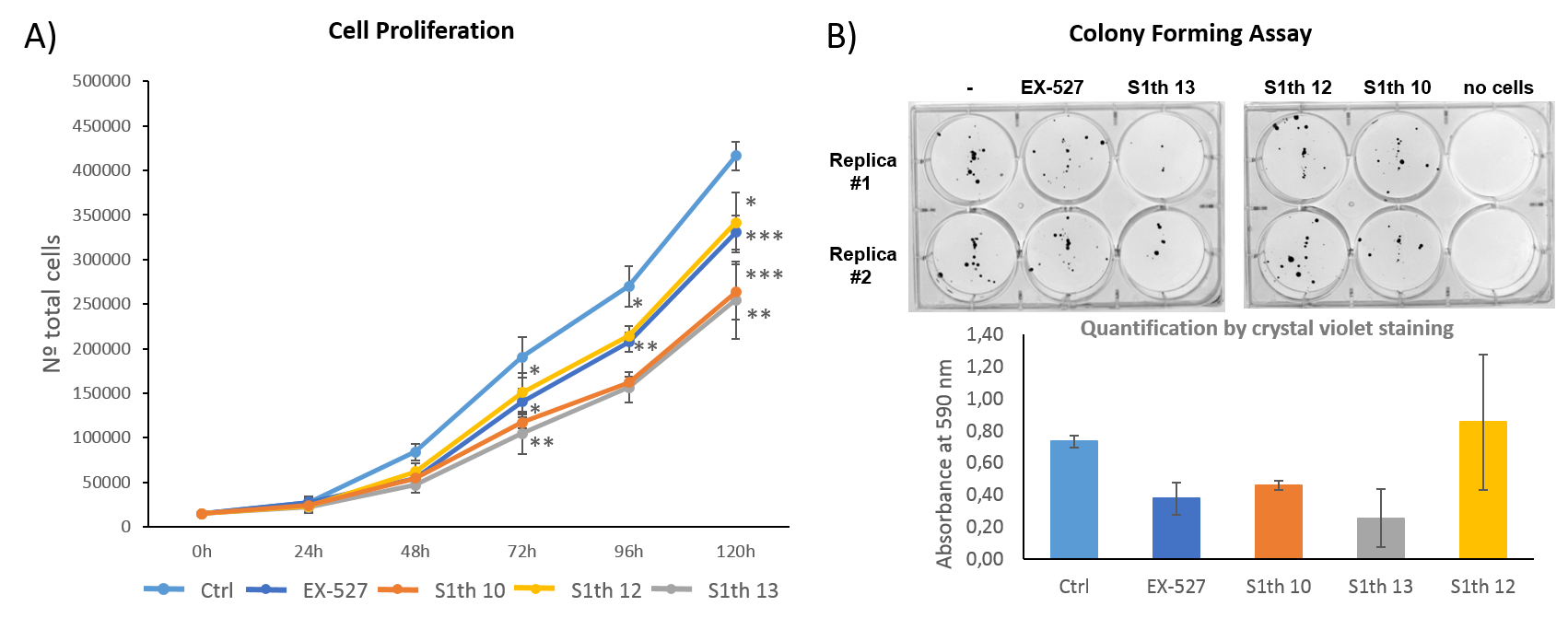


**Supplementary Figure 5.** Cell proliferation and colony forming capabilities of MCF7 cells after treatment with the S1th or EX-527. A) Impact of **S1th 10**, **12** and **13** and EX-527 on cell proliferation of MCF7 cells. All compounds are able to slow down cell proliferation of MCF7 cells, with **S1th 13** and **S1th 10** being most effective, while EX-527 and **S1th 12** only have a mild effect. The experiments were performed at least three times each. * (p < 0.05), ** (p < 0.01), *** (p < 0.001). B) Impact of EX-527 and **S1th 10**, **12** and **13** on colony forming capabilities of MCF7 cells. Colony forming is significantly decreased by EX-527 and **S1th 13**, slightly decreased by **S1th 10** but barely affected by **S1th 12**. Experiments were carried out in duplicates and quantification was done by staining the colonies with crystal violet and measuring absorbance at 590 nm.

**Supplementary Table 2.** Primary screen of suggested compounds for sirtuin inhibition. All compounds are available at Princeton BioMolecular Research.

| Compound | Inhibition @ 50 µM | | | Compound | Inhibition @ 50 µM | | |
| --- | --- | --- | --- | --- | --- | --- | --- |
|  | Sirt1 | Sirt2 | Sirt3 |  | Sirt1 | Sirt2 | Sirt3 |
| OSSK 925126 | n.i. | 16% | n.i. | OSSK 987000 | n.i. | n.i. | n.i. |
| OSSK 984008 | n.i. | n.i. | n.i. | OSSK 921836 | n.i. | n.i. | n.i. |
| OSSK 677292 | n.i. | 17% | n.i. | OSSK 713772 | n.i. | n.i. | n.i. |
| OSSK 660153 | n.i. | n.i. | n.i. | OSSK 444962 | n.i. | n.i. | n.i. |
| OSSK 659424 | n.i. | n.i. | n.i. | OSSK 360152 | n.i. | n.i. | n.i. |
| OSSK 628038 | n.i. | n.i. | n.i. | OSSK 337337 | n.i. | n.i. | n.i. |
| OSSK 628068 | n.i. | n.i. | n.i. | OSSK 310299 | n.i. | n.i. | n.i. |
| OSSK 567109 | n.i. | n.i. | n.i. | OSSK 280440 | n.i. | n.i. | n.i. |
| OSSK 854972^**^ | n.i. | n.i. | n.i. | OSSK 363499 | n.i. | n.i. | n.i. |
| OSSK 401049 | n.i. | n.i. | n.i. | OSSK 342532 | n.i. | 16% | n.i. |
| OSSK 478639 | n.i. | 15% | n.i. | OSSK 318655 | n.i. | n.i. | n.i. |
| OSSK 515470 | n.i. | n.i. | n.i. | OSSL 309945 | n.i. | 29% | n.i. |
| OSSK 515461 | n.i. | 26% | n.i. | OSSL 294309 | n.i. | 42% | n.i. |
| OSSK 373794 | n.i. | 45% | n.i. | OSSL 105797 | n.i. | n.i. | n.i. |
| OSSK 998596 | n.i. | 29% | n.i. | OSSK 921921 | n.i. | n.i. | n.i. |
| OSSK 660579 | n.i. | n.i. | n.i. | OSSK 671780 | n.i. | 12% | n.i. |
| OSSL 041990 | 17% | 36% | n.i. | OSSK 671324 | n.i. | n.i. | n.i. |
| OSSL 001450 | n.i. | n.i. | n.i. | OSSK 595686 | n.i. | n.i. | n.i. |
| OSSL 266644 | n.i. | n.i. | n.i. | OSSK 571792 | n.i. | n.i. | n.i. |
| OSSL 001504 | n.i. | 24% | n.i. | OSSK 544893 | n.i. | n.i. | n.i. |
| OSSK 017079 | n.i. | n.i. | n.i. | OSSK 393678 | n.i. | n.i. | n.i. |
| OSSK 285614 | n.i. | n.i. | n.i. | OSSK 444680 | n.i. | n.i. | n.i. |
| OSSK 026091 | n.i. | n.i. | n.i. | OSSK 480394 | 49% | 29% | n.i. |
| OSSK 014373^*^ | 16% | 41% | n.i. | OSSK 530294 | n.i. | n.i. | n.i. |
| OSSK 359892 | n.i. | n.i. | n.i. | OSSK 537984 | n.i. | n.i. | n.i. |
| OSSK 222160 | n.i. | n.i. | n.i. | OSSK 538452 | n.i. | 17% | n.i. |
| OSSK 221646 | 82% | 23% | n.i. | OSSK 705741 | n.i. | n.i. | n.i. |
| OSSL 308099 | n.i. | n.i. | n.i. | OSSK 706267 | n.i. | 17% | n.i. |
| OSSL 075801 | n.i. | n.i. | n.i. | OSSK 715319 | 19% | n.i. | n.i. |
| OSSK 587243 | n.i. | 12% | n.i. | OSSK 766725 | n.i. | n.i. | n.i. |
| OSSL 280987 | n.i. | 11% | n.i. | OSSK 766798 | n.i. | 19% | n.i. |
| OSSL 266012 | n.i. | 47% | n.i. | OSSK 776970 | 20% | 11% | n.i. |
| OSSL 117189 | 15% | 16% | n.i. | OSSK 979070 | n.i. | 35% | 23% |
| OSSL 125753 | 60% | 12% | n.i. | OSSK 980849 | n.i. | n.i. | n.i. |
| OSSL 259882 | n.i. | n.i. | n.i. | OSSK 705764 | n.i. | n.i. | n.i. |
| OSSL 117187 | n.i. | 13% | n.i. |  |  |  |  |

n.i. = no inhibition (<10 %)

^*^ autofluorescent

^**^ highly autofluorescent, interferes with assay signal
